# Case Report: Diagnostic anchoring in spinal dural arteriovenous fistula: a case-based analysis of clinical-radiological mismatch and delayed recognition

**DOI:** 10.3389/fmed.2026.1845311

**Published:** 2026-07-31

**Authors:** Xiaobin Luo, Yali Zhong, Cong Hou, Yong Zou, Hao Ding, Chenghao Yang

**Affiliations:** 1 Department of Neurosurgery, Zigong Fourth People’s Hospital, Zigong, Sichuan, China; 2 Department of Neurosurgery, The Affiliated Hospital of Southwest Medical University, Luzhou, Sichuan, China; 3 Department of Gastroenterology, Zigong First People’s Hospital, Zigong, Sichuan, China

**Keywords:** case report, digital subtraction angiography, inflammatory myelopathy, lumbar degenerative disease, spinal dural arteriovenous fistula

## Abstract

Spinal dural arteriovenous fistula (SDAVF) is an uncommon but treatable cause of progressive myelopathy, and its diagnosis is frequently delayed because early symptoms may be misattributed to lumbar degenerative disease, inflammatory myelopathy, or peripheral neurological disorders. We report a 49-year-old man with progressive bilateral lower-limb weakness and numbness over 1 week, followed by marked deterioration within 2 days. Initial lumbar imaging showed disc bulging and degenerative changes, creating a potential diagnostic anchor; however, the bilateral deficits, rapid progression, gait impairment, and urinary and bowel dysfunction were disproportionate to the lumbar findings. Thoracic magnetic resonance imaging (MRI) demonstrated longitudinal intramedullary T2 hyperintensity extending from T5 to T12 and suspected dorsal serpiginous perimedullary flow voids, suggesting venous congestive myelopathy caused by a spinal vascular lesion. Although inflammatory or demyelinating myelopathy was considered, cerebrospinal fluid examination was deferred at that stage because dorsal perimedullary flow voids and the overall imaging pattern made a vascular etiology more likely and more urgent to confirm. Digital subtraction angiography (DSA) was therefore prioritized and confirmed a right T5 SDAVF. The patient underwent microsurgical disconnection, with postoperative angiography confirming complete obliteration and gradual neurological improvement thereafter. The educational value of this case lies not in a novel imaging feature or treatment strategy, but in illustrating how sequential diagnostic anchoring can delay recognition of SDAVF and how clinical-radiological mismatch should prompt timely angiographic evaluation in patients with rapidly progressive myelopathy.

## 1 Introduction

Spinal dural arteriovenous fistula (SDAVF) is a rare but potentially serious spinal vascular malformation that can lead to progressive venous congestive myelopathy. Its exact pathogenesis remains incompletely understood; however, previous studies suggest that venous hypertension may play a critical role in the development and progression of SDAVF-related neurological symptoms (1). The incidence of SDAVF shows sex-related differences and increases with age, particularly in male patients, which partly explains why it is often encountered in middle-aged and older populations with coexisting degenerative spinal changes (2). Despite these observations, early diagnosis of SDAVF remains challenging in routine clinical practice.

As the disease progresses, patients may develop gait disturbance, lower-limb weakness, sensory symptoms, and autonomic dysfunction, including bladder or bowel disturbances. These manifestations frequently overlap with those of more common degenerative spinal disorders, leading to diagnostic anchoring on lumbar degenerative disease, particularly when imaging reveals disc bulging or spinal stenosis. However, such findings are common and may not fully explain the clinical presentation, especially when symptoms are bilateral, rapidly progressive, or accompanied by urinary or bowel dysfunction. In addition, longitudinal spinal cord T2 hyperintensity may raise concern for inflammatory or demyelinating myelopathy, further complicating the diagnostic process. Misdiagnosis and delayed recognition of SDAVF have been widely reported and may result in irreversible neurological deficits (3, 4).

In recent years, advances in imaging techniques have improved the diagnosis of SDAVF. Magnetic resonance imaging (MRI) is commonly used as an initial diagnostic modality and may demonstrate spinal cord edema and perimedullary flow voids, suggestive of venous congestive myelopathy. Magnetic resonance angiography (MRA) has also been shown to be a sensitive tool for localizing the fistula and may help reduce the false-negative rate of digital subtraction angiography (DSA) in selected cases (5). Nevertheless, DSA remains the gold standard for definitive diagnosis because it can confirm the presence of a fistula, identify the arterial feeder, and guide treatment planning. Both endovascular and surgical approaches are available for treatment, and embolization using agents such as n-butyl cyanoacrylate (nBCA) or Onyx has been widely applied in recent years (6). When complete obliteration of the fistula is required, microsurgical disconnection remains the most reliable and commonly used approach (7).

In this report, we describe a patient with rapidly progressive myelopathy whose initial evaluation was influenced by lumbar degenerative findings, while the short clinical course also raised concern for inflammatory myelopathy. Rather than presenting the case as a novel disease manifestation, imaging feature, or treatment strategy, we use it as a case-based analysis of diagnostic failure and clinical reasoning. This case highlights the process of sequential diagnostic anchoring, from lumbar degenerative disease to inflammatory myelopathy, before a spinal vascular lesion was considered. It also emphasizes the importance of recognizing clinical-radiological mismatch and prioritizing DSA when bilateral lower-limb symptoms, urinary or bowel dysfunction, and spinal MRI flow voids cannot be explained by lumbar degenerative findings alone.

## 2 Case description

### 2.1 Patient information

A 49-year-old man was admitted with a 1-week history of progressive weakness and numbness in both lower limbs. During the 2 days before admission, his symptoms had worsened markedly, and he developed significant difficulty walking.

At symptom onset, approximately 1 week before admission, the patient noticed numbness over the anterior aspect of the left thigh and mild weakness in both lower limbs, without an obvious precipitating factor. He was initially still able to walk with a limp and had not received regular treatment.

Before referral to our institution, he had been evaluated at a local hospital. Lumbar computed tomography revealed disc bulging and degenerative bony changes, which were initially considered a possible explanation for his symptoms. However, no specific treatment was initiated, and his neurological symptoms continued to progress. The clinical timeline is summarized in Table 1.

**TABLE 1:** Timeline of clinical events.

Time point	Clinical course and management
1 week before admission	Onset of numbness over the anterior left thigh and mild bilateral lower limb weakness without an obvious precipitating factor; the patient could walk with a limp and received no regular treatment.
Following days	Progressive bilateral lower-limb weakness and numbness became more apparent.
2 days before admission	Marked worsening of symptoms with difficulty walking.
At admission	Outside lumbar computed tomography showed disc bulging and degenerative changes, which were initially considered relevant to the symptoms; urinary difficulty, constipation, and difficulty with defecation were also reported.
Further diagnostic work-up	Because the lumbar findings did not fully explain the bilateral symptoms, urinary and bowel dysfunction, and rapid progression, a thoracolumbar MRI was performed, which showed long-segment intramedullary signal change from T5 to T12 with dorsal flow voids. Inflammatory myelopathy was considered, but cerebrospinal fluid examination was deferred at that stage because dorsal perimedullary flow voids and the overall MRI pattern favored a vascular etiology; DSA confirmed SDAVF at the right T5 level.
Surgical intervention	Right T5 laminectomy and microsurgical disconnection of the fistula were performed, followed by angiographic confirmation of obliteration.
Follow-up	Postoperative monitoring showed clinical stability; lower-limb strength and sensory symptoms gradually improved, and lower-limb strength had recovered to grade 5/5 by discharge.

### 2.2 Clinical findings

On admission, neurological examination showed decreased sensation over the anterior aspect of the left thigh. Proximal lower limb strength and ankle dorsiflexion were largely preserved, whereas bilateral great-toe dorsiflexion and ankle plantar flexion were mildly reduced, corresponding to Medical Research Council grade 4/5.

No pathological reflexes were elicited, and Babinski signs were negative bilaterally. Saddle-area sensation was preserved. The patient also reported urinary difficulty, constipation, and difficulty with defecation, which had developed in parallel with the worsening of motor symptoms.

The overall clinical pattern suggested involvement beyond a single lumbar nerve-root distribution. In particular, the bilateral symptoms, rapid progression, gait impairment, and urinary and bowel dysfunction raised concern for a myelopathic process rather than isolated lumbar degenerative disease.

### 2.3 Diagnostic assessment

Lumbar computed tomography showed disc bulging and degenerative changes. Although these findings initially appeared clinically relevant, they did not adequately explain the bilateral distribution of symptoms, rapid neurological deterioration, urinary and bowel dysfunction, or the extent of gait impairment. This represented an important clinical-radiological mismatch and prompted further evaluation with thoracolumbar spinal MRI.

**FIGURE 1:**
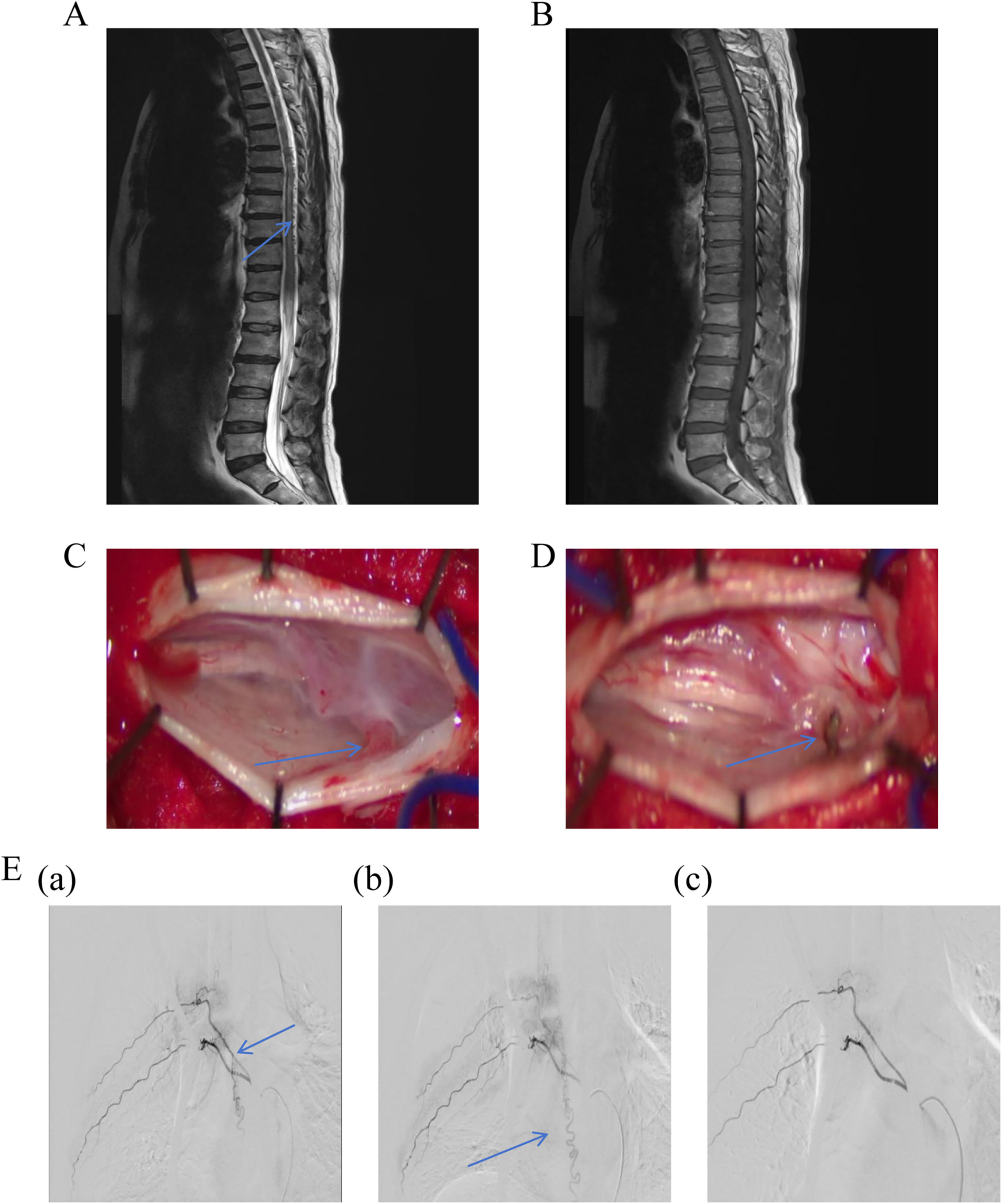
Radiological and intraoperative findings of spinal dural arteriovenous fistula (SDAVF). **(A)** Preoperative sagittal T2-weighted MRI showing longitudinal intramedullary T2 hyperintensity in the thoracic spinal cord and suspected dorsal serpiginous perimedullary flow voids (arrow), suggestive of venous congestive myelopathy. **(B)** Gadolinium-enhanced sagittal T1-weighted MRI of the thoracic spine, obtained as part of the preoperative evaluation. **(C)** Intraoperative view showing dilated and arterialized draining veins on the dorsal surface of the spinal cord (arrow). **(D)** Intraoperative view after disconnection of the fistula, demonstrating collapse of the abnormal draining vein (arrow). **(E)** Digital subtraction angiography (DSA): (a) Preoperative angiography revealing abnormal arteriovenous shunting at the right T5 level (arrow); (b) early venous drainage through dilated veins (arrow); (c) postoperative angiography confirming complete obliteration of the fistula.

Thoracic spinal magnetic resonance imaging demonstrated longitudinal intramedullary T2 hyperintensity extending from T5 to T12, together with dorsal serpiginous perimedullary flow voids on sagittal T2-weighted imaging (Figure 1A). Gadolinium-enhanced sagittal T1-weighted MRI was also obtained as part of the preoperative evaluation (Figure 1B). These findings, together with the progressive bilateral deficits and autonomic dysfunction, suggested venous congestive myelopathy caused by a spinal vascular lesion, particularly SDAVF.

Inflammatory or demyelinating myelopathy remained a relevant differential diagnosis because of the subacute clinical deterioration and longitudinal spinal cord signal abnormality. However, cerebrospinal fluid examination was deferred at this stage because the MRI pattern, especially the dorsal perimedullary flow voids, strongly favored a vascular etiology. Digital subtraction angiography was therefore prioritized because it could definitively confirm the diagnosis, localize the fistula, and guide timely treatment planning. DSA demonstrated abnormal arteriovenous shunting at the right T5 level, confirming the diagnosis of SDAVF [Figure 1E (a, b)].

### 2.4 Diagnostic reasoning and decision-making

The diagnostic process in this case involved several sequential steps. First, the presence of lumbar disc bulging and degenerative changes created a potential diagnostic anchor, as these findings are common in middle-aged patients with lower-limb symptoms. However, the clinical presentation was not fully consistent with isolated lumbar degenerative disease, because the symptoms were bilateral, rapidly progressive, and accompanied by urinary and bowel dysfunction. Second, inflammatory or demyelinating myelopathy was considered because the patient deteriorated over a short period, and MRI showed longitudinal intramedullary T2 hyperintensity. Nevertheless, the presence of dorsal serpiginous flow voids shifted the diagnostic focus toward a vascular cause. Third, the combination of clinical-radiological mismatch, longitudinal cord edema, and perimedullary flow voids supported venous congestive myelopathy due to SDAVF. On this basis, DSA was prioritized over cerebrospinal fluid examination to avoid delaying definitive vascular diagnosis and treatment.

### 2.5 Therapeutic intervention

The patient underwent microsurgical treatment consisting of right T5 laminectomy and disconnection of the fistula. The purpose of surgery was to eliminate the abnormal arteriovenous shunt and relieve venous congestion of the spinal cord.

Intraoperatively, dilated and arterialized draining veins were observed on the dorsal surface of the spinal cord (Figure 1C). After disconnection of the fistula, the draining vein collapsed, indicating successful interruption of the abnormal shunt (Figure 1D).

### 2.6 Follow-up and outcomes

Postoperative DSA confirmed complete obliteration of the fistula, with no residual abnormal vascular flow [Figure 1E (c)]. In the early postoperative period, the patient was monitored in the intensive care unit and remained clinically stable. His neurological function gradually improved after surgery. Lower-limb strength recovered, sensory symptoms were alleviated, and urinary and bowel symptoms improved. By discharge, lower-limb muscle strength had improved to Medical Research Council grade 5/5, and the surgical wound had healed well.

## 3 Discussion

An abnormal communication between a dural branch of a radicular artery and a radicular vein characterizes SDAVF. This abnormal shunt results in arterialization of the venous system and a subsequent increase in spinal venous pressure (8). Because spinal veins are valveless, elevated venous pressure can be transmitted along the perimedullary venous plexus, thereby reducing the arteriovenous pressure gradient, impairing normal venous drainage, and producing venous congestion of the spinal cord. Over time, this process may lead to vasogenic edema, intramedullary T2 hyperintensity, progressive myelopathy, and, in advanced cases, ischemic injury (6). The severity and tempo of clinical manifestations may vary according to shunt flow, venous outflow capacity, collateral drainage, and the duration of venous hypertension. This pathophysiological process explains why SDAVF often presents with insidious and progressive neurological deficits, but may also deteriorate rapidly when venous drainage becomes decompensated.

In clinical practice, SDAVF remains difficult to recognize, particularly in its early stages. Presenting symptoms are often nonspecific and may include lower-limb weakness, sensory disturbances, gait instability, and, in more advanced cases, bladder or bowel dysfunction (9). These symptoms overlap with those of more common degenerative spinal disorders, peripheral neuropathies, and inflammatory or demyelinating myelopathies. As a result, SDAVF may not be considered early in the diagnostic process, especially when lumbar imaging shows degenerative abnormalities that appear to offer a convenient explanation for the patient’s symptoms.

The present case illustrates this diagnostic vulnerability. Lumbar disc bulging and degenerative bony changes initially created a potential diagnostic anchor. However, the patient’s bilateral lower-limb symptoms, rapid progression, gait impairment, urinary and bowel dysfunction, and long-segment thoracic cord T2 hyperintensity were not adequately explained by isolated lumbar radiculopathy or degenerative lumbar disease. The short clinical course and longitudinal spinal cord signal abnormality also made inflammatory or demyelinating myelopathy a reasonable differential diagnosis. Thus, the diagnostic process moved sequentially from lumbar degenerative disease to inflammatory myelopathy before a spinal vascular lesion was recognized.

**FIGURE 2:**
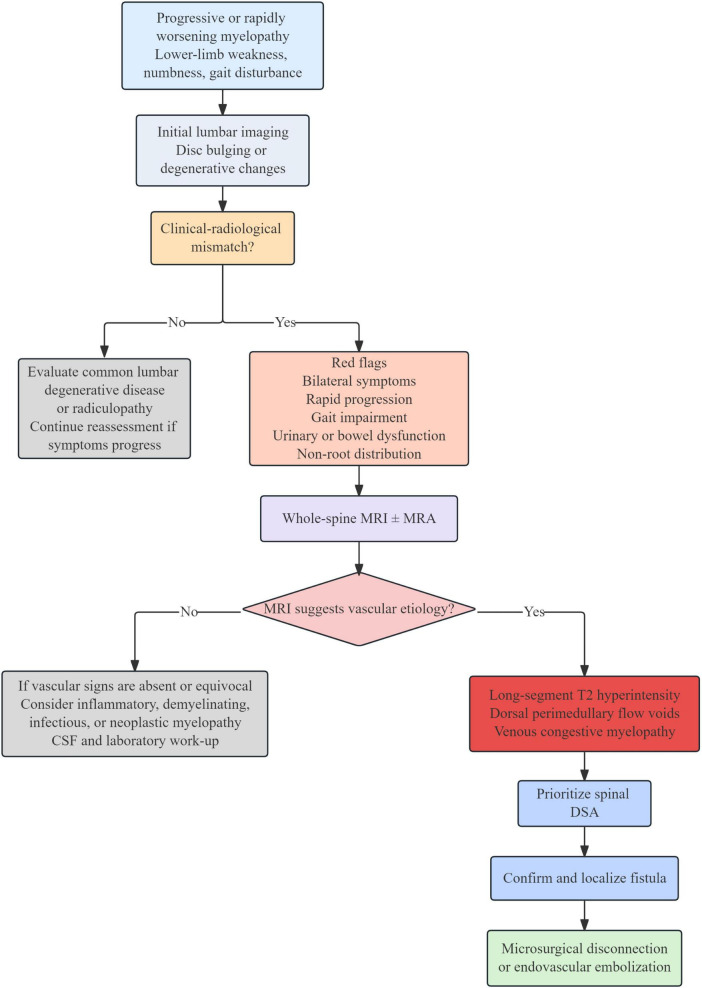
Proposed diagnostic framework for rapidly progressive myelopathy with suspected spinal dural arteriovenous fistula (SDAVF). In patients presenting with progressive or rapidly worsening myelopathy, lumbar degenerative findings should be interpreted cautiously when they do not adequately explain bilateral lower-limb symptoms, rapid progression, gait impairment, or urinary and bowel dysfunction. This clinical-radiological mismatch should prompt whole-spine MRI. Long-segment intramedullary T2 hyperintensity, spinal cord edema, and dorsal perimedullary flow voids suggest venous congestive myelopathy and should lead to timely digital subtraction angiography (DSA) for definitive diagnosis and treatment planning. CSF, cerebrospinal fluid; MRI, magnetic resonance imaging; MRA, magnetic resonance angiography.

This sequence highlights the importance of clinical-radiological mismatch. Delayed or incorrect diagnosis is common in SDAVF because early symptoms are frequently attributed to more common disorders. Takai and Taniguchi reported that 31 of 40 patients with SDAVF were initially misdiagnosed, most commonly as lumbar spinal stenosis or other nonvascular spinal disorders (10). A recent scoping review including 18 studies and 965 SDAVF cases further showed that misdiagnosis rates ranged from 17.5% to 100%, with an average interval of approximately 10–15 months from early symptoms to confirmed diagnosis (11). These findings suggest that diagnostic delay is driven not only by the rarity of SDAVF, but also by premature diagnostic anchoring on more familiar conditions when progressive lower-limb symptoms and sphincter dysfunction are present. Previous reports indicate that, across different mimicking diagnoses, the key missed clues are often similar: bilateral or ascending lower-limb symptoms, gait impairment disproportionate to lumbar imaging findings, early urinary or bowel dysfunction, longitudinal spinal cord T2 hyperintensity, and dorsal perimedullary flow voids. These recurring diagnostic pitfalls formed the basis for the diagnostic clues summarized in Table 2 and the proposed clinical framework shown in Figure 2.

**TABLE 2:** Diagnostic clues and decision-making considerations distinguishing SDAVF from common mimicking disorders.

Differential diagnosis	Why it may be initially considered	Features prompting diagnostic reconsideration	Implication in the present case
Lumbar degenerative disease or radiculopathy	Lumbar CT showed disc bulging and degenerative bony changes; lower-limb numbness and weakness may initially resemble lumbar radiculopathy or lumbar spinal stenosis.	Bilateral and rapidly progressive symptoms, gait impairment, urinary difficulty, constipation, and defecation difficulty were disproportionate to the lumbar imaging findings; the deficits were not confined to a single nerve-root distribution.	Lumbar degenerative findings were interpreted as a potential diagnostic anchor rather than a sufficient explanation for the full clinical picture, prompting further evaluation with thoracolumbar spinal MRI.
Inflammatory or demyelinating myelopathy	Subacute deterioration and longitudinal intramedullary T2 hyperintensity may raise concern for inflammatory myelopathy, NMOSD, MOGAD-associated myelopathy, or idiopathic transverse myelitis.	Dorsal serpiginous perimedullary flow voids, progressive bilateral deficits, and autonomic dysfunction favored venous congestive myelopathy; absence of fever or meningeal signs alone was not used to exclude inflammatory disease.	Inflammatory or demyelinating myelopathy remained a relevant differential diagnosis, but vascular MRI features shifted the diagnostic priority toward SDAVF and DSA. CSF and laboratory work-up would be appropriate if vascular signs were absent or uncertain.
Peripheral neuropathy, Guillain-Barré-like presentation, or cauda equina syndrome	Lower-limb weakness, sensory symptoms, impaired mobility, and bladder or bowel complaints may initially resemble peripheral nerve, polyradicular, or cauda equina disorders.	Long-segment thoracic cord T2 hyperintensity and dorsal perimedullary flow voids indicated a central spinal cord process with venous congestive features rather than a purely peripheral disorder.	Peripheral neurological disorders became less likely after thoracolumbar MRI demonstrated spinal cord involvement and vascular flow-void abnormalities.
Spinal dural arteriovenous fistula / venous congestive myelopathy	Progressive bilateral lower-limb symptoms, gait impairment, urinary and bowel dysfunction, long-segment intramedullary T2 hyperintensity from T5 to T12, and dorsal perimedullary flow voids were compatible with SDAVF.	Early symptoms may be nonspecific, and coexisting lumbar degenerative findings may obscure recognition of the spinal vascular lesion.	The combination of clinical-radiological mismatch and vascular MRI signs justified prioritizing DSA, which confirmed a right T5 SDAVF and guided treatment planning.

SDAVF, spinal dural arteriovenous fistula; CT, computed tomography; MRI, magnetic resonance imaging; DSA, digital subtraction angiography; CSF, cerebrospinal fluid; NMOSD, neuromyelitis optica spectrum disorder; MOGAD, myelin oligodendrocyte glycoprotein antibody-associated disease.

Magnetic resonance imaging (MRI) provides important clues for identifying SDAVF. Typical findings include spinal cord edema and serpiginous flow voids along the dorsal aspect of the cord, reflecting dilated perimedullary veins. However, these features may be subtle, overlooked, or misinterpreted as nonspecific changes. In the present case, the long-segment intramedullary signal abnormality extending from T5 to T12, together with dorsal serpiginous flow voids, was a key imaging clue suggesting venous congestive myelopathy caused by a spinal vascular lesion. For non-neurosurgical clinicians, bilateral or ascending lower-limb symptoms, gait impairment out of proportion to lumbar imaging findings, early urinary or bowel dysfunction, long-segment cord edema, and dorsal flow voids should prompt consideration of SDAVF and timely angiographic evaluation.

Digital subtraction angiography (DSA) remains the gold standard for diagnosing SDAVF because it allows precise visualization of the vascular anatomy, identification of the feeding artery and draining vein, and accurate localization of the fistula (9, 12). In the present case, cerebrospinal fluid examination was deferred not because inflammatory myelopathy was impossible, but because the combination of clinical-radiological mismatch and dorsal perimedullary flow voids made a vascular etiology more likely and more urgent to confirm. DSA was therefore prioritized because it could establish the diagnosis, localize the lesion, and guide definitive treatment planning. Postoperative angiographic assessment also played an essential role in confirming complete obliteration of the fistula (13).

From a differential diagnostic perspective, SDAVF should be distinguished from lumbar degenerative disease, peripheral neuropathy or polyradicular disorders, and inflammatory or demyelinating myelopathy (14, 15). In patients initially suspected of having lumbar degenerative disease, symptoms confined to a single nerve-root distribution may support radiculopathy, whereas bilateral or ascending progression, gait disturbance, urinary or bowel dysfunction, or thoracic cord signal abnormalities should prompt diagnostic reassessment. In patients suspected of having inflammatory or demyelinating myelopathy, longitudinal intramedullary T2 hyperintensity may be misleading; however, dorsal serpiginous perimedullary flow voids, progressive venous congestive features, and autonomic dysfunction disproportionate to other findings should raise concern for SDAVF. This distinction is clinically important because delayed recognition may permit ongoing venous congestion and irreversible neurological injury, whereas timely fistula obliteration can halt further progression. The diagnostic clues summarized in Table 2 are therefore intended to complement the proposed clinical framework shown in Figure 2.

Another aspect worth noting is the clinical course of SDAVF, which is often progressive but not strictly linear (16, 17). Although the present patient had only a short recognized symptomatic course, his condition changed from mild gait disturbance to marked walking difficulty within several days. This rapid deterioration may reflect decompensation of a previously compensated venous drainage system, increasing venous pressure, reduced collateral outflow, or transient changes in intrathoracic and intra-abdominal pressure (18). In the available history, no obvious precipitating factor was identified. Therefore, the recent worsening was considered most likely related to progression from compensated venous congestion to clinically overt myelopathy.

Regarding treatment, both endovascular and surgical approaches have been described (19). Microsurgical disconnection of the fistula is widely considered an effective and definitive treatment, with high success rates (20). In this case, surgical disconnection resulted in collapse of the arterialized draining vein, and postoperative angiographic assessment confirmed complete obliteration of the fistula. The patient’s gradual postoperative neurological improvement was consistent with relief of venous congestion after successful interruption of the abnormal shunt.

The main value of this report is educational rather than novel in disease mechanism, imaging appearance, or treatment strategy. The case demonstrates a common diagnostic failure pattern in which progressive myelopathy is initially interpreted through more familiar diagnoses, including lumbar degenerative disease and inflammatory myelopathy, before vascular clues are recognized. By integrating the patient’s clinical course with previous misdiagnosis literature, this report emphasizes three practical lessons: first, degenerative lumbar findings should not be accepted as explanatory when symptoms are bilateral, rapidly progressive, or accompanied by sphincter dysfunction; second, long-segment cord edema with dorsal flow voids should prompt suspicion of venous congestive myelopathy; and third, DSA should be prioritized when MRI findings suggest SDAVF because it provides definitive diagnosis and treatment guidance. This report has limitations. The proposed diagnostic framework is derived from a single case and relevant misdiagnosis literature, and it has not been prospectively validated. Therefore, it should be interpreted as an educational aid for clinical reasoning rather than a formal diagnostic guideline.

Overall, this case reinforces the importance of integrating clinical judgment with imaging findings in patients with progressive or rapidly worsening myelopathy. Early recognition of clinical-radiological mismatch is essential for avoiding diagnostic delay, enabling timely angiographic evaluation, and preventing irreversible neurological damage.

## 4 Conclusions

SDAVF should be considered in patients with progressive or rapidly worsening myelopathy, particularly when lumbar imaging findings do not adequately explain bilateral lower-limb symptoms, gait impairment, or urinary and bowel dysfunction. This case highlights how diagnostic anchoring on lumbar degenerative disease or inflammatory myelopathy may delay recognition of a spinal vascular lesion. Careful clinical-radiological correlation, attention to long-segment cord edema and dorsal perimedullary flow voids on MRI, and timely prioritization of angiographic evaluation are essential for early diagnosis and appropriate treatment.

## Data Availability

The original contributions presented in the study are included in the article/supplementary material, further inquiries can be directed to the corresponding authors.
